# Associations Between Nurse Staffing Levels and 30‐ and 60‐Day Readmissions for Acute Care Patients With Intellectual and Developmental Disability

**DOI:** 10.1002/nur.70013

**Published:** 2025-08-11

**Authors:** Lynne S. Moronski, Eileen T. Lake, Matthew D. McHugh

**Affiliations:** ^1^ Center for Health Outcomes and Policy Research University of Pennsylvania School of Nursing Philadelphia PA USA; ^2^ Leonard Davis Institute for Health Economics University of Pennsylvania Philadelphia PA USA

**Keywords:** bias, delivery of health care, developmental disabilities, nurses, patient discharge, quality of health care, workforce, working conditions

## Abstract

The purpose of this study was to examine the association between hospital nurse staffing levels and 30‐ and 60‐day readmissions among patients with intellectual and/or developmental disabilities (IDD). This cross‐sectional correlational study utilized secondary data from 595 acute care, nonfederal hospitals in California, Florida, New Jersey, and Pennsylvania in 2016. Data were obtained from three sources: the American Hospital Association Annual Survey, the RN4CAST‐US nurse survey, and state patient hospital discharge summaries. The analytic sample included 39,558 hospital stays for 28,446 adults with IDD aged 18 and older who were discharged alive. In adjusted models, every additional patient added to a nurse's workload was associated with 7% higher odds of 30‐day readmission OR = 1.07 (95% CI [1.03, 1.12], *p* = 0.001) and 9% higher odds of 60‐day readmission OR = 1.09 (95% CI [1.04, 1.13], *p* < 0.001) among patients with IDD. The average nurse staffing level across hospitals was 4.7 patients per nurse (SD = 0.99). Staffing levels varied by hospital characteristics, with large hospitals, major teaching hospitals, and hospitals in California having better staffing ratios. The study population had a 30‐day readmission rate of 17.1%, which is 27% higher than the average adult hospital 30‐day readmission rate in the US in 2018. This study demonstrates the association of nurse staffing levels with readmission odds for patients with IDD. Improving nurse staffing levels is a system‐based solution that can potentially improve outcomes for patients with IDD, who often require intensive nursing care.

## 1 Introduction

According to the World Health Organization, over one billion people around the world live with a disability (World Bank 2023). In the United States, more than 2.28 million adults are estimated to have an intellectual and/or developmental disability (IDD) (University of Minnesota 2024). The American Association of Intellectual and Developmental Disabilities defines intellectual disability (ID) as a condition involving cognitive limitations, typically reflected by an IQ of approximately 70. Additionally, an ID diagnosis requires substantial challenges in at least two adaptive functioning domains, such as communication, personal care, or social interaction, which typically manifest before age 22 (American Association on Intellectual and Developmental Disabilities 2025).

Developmental disabilities (DD) encompass a broader range of severe and chronic conditions resulting from mental and/or physical impairments, such as autism spectrum disorder (Centers for Disease Control and Prevention 2024). The overlap is significant: while all individuals with ID are considered to have DD, not all those with DD meet the criteria for ID (Integrity Inc. 2019). For example, autism spectrum disorder is a developmental disorder, but can also be considered an intellectual disability when IQ is 70 or lower. Both ID and DD are often grouped (IDD) due to the significant impact on both adaptive behavior and functioning across the lifespan.

People with IDD face significant lifelong health disparities. In 2023, the National Institutes of Health designated people with IDD as a health disparity population (Perez‐Stable and Valdez 2023). Compared to the general population, individuals with IDD often experience higher rates of comorbidities, worse overall health, and earlier onset of age‐related decline due to their complex health needs across the lifespan. These disparities are worsened by limited access to preventative care, healthcare system barriers, provider and staff biases, and higher mortality rates (Ervin and Merrick 2014).

People living with IDD are more likely than the general population to have comorbidities, often in combination, such as obesity, diabetes, cardiovascular disease (Escudé 2022), epilepsy, gastrointestinal reflux, infectious disease, heart disease, musculoskeletal disease, diabetes, and mental illness (Reppermund et al. 2020). Multimorbidity is associated with a higher risk of mortality and greater use of healthcare services, including hospitalization (Ervin and Merrick 2014). Estimates suggest that the rate of hospitalization is as much as three times as high for people with IDD than those in age‐ and sex‐matched control groups (Ailey et al. 2014; Hosking et al. 2017; Lindgren et al. 2021). Even after controlling for other characteristics (e.g., other comorbidities), IDD persists as a significant predictor of readmissions (Balogh et al. 2018). Mental illness presents an ongoing challenge because it appears more frequently in those with IDD and has been associated with a 1.7 times more likely 30‐day hospital readmission (Balogh et al. 2018).

Various strategies have been proposed to address these disparities. Individual‐level approaches include tailored IDD inpatient programs (Wirtz et al. 2020) and nurse liaison positions in England designed to enhance support and communication for patients with IDD while also providing targeted education to hospital staff (Appelgren et al. 2018; Moloney et al. 2021; Redley et al. 2019). Preadmission support, such as hospital tours and introductions to the involved healthcare professionals, were offered to patients with IDD before admission (Moloney et al. 2021; Webber et al. 2010). In one case, the patient with IDD was visited and sedated at home by the anesthesiologist and surgeon before hospital transfer (Heslop et al. 2019; Moloney et al. 2021). While such programs have been associated with reduced length of stay and lower healthcare costs, there was no association between the special programs and a reduction in 30‐day readmissions.

An example of a system‐level approach includes nurse staffing levels. Nurses provide patient care around the clock in acute care hospitals. It is crucial to maintain manageable workloads to ensure that nurses can efficiently and reliably provide and coordinate patient care, anticipate clinical deterioration, stop harm, and educate patients and their families (McHugh et al. 2021). Over 20 years of evidence have demonstrated that every additional patient per nurse is associated with worse patient outcomes, including increased mortality, infections, and adverse events (Aiken 2002; Haegdorens et al. 2019). Nurse staffing levels have also been linked directly to hospital readmissions (Ma et al. 2014) — a common experience for patients with IDD.

This study is informed by the Quality Health Outcomes Model (QHOM), which posits a systems approach, linking systems, interventions, client (patient) characteristics, and outcomes (Mitchell et al. 1998). Within this framework, systems include hospital‐level attributes, with differentiations in size, technology, teaching status (i.e., residents and fellows) and nursing features (e.g., nurse staffing levels). Patient characteristics, including IDD status, social determinants of health, comorbidities, and reasons for admission, are considered client characteristics in the QHOM model. The QHOM model is unique because it does not assume a direct path from intervention to outcome, despite multiple feedback loops that delineate the association between nursing structure and interventions on patient outcomes. Instead, the effects of the intervention indirectly depend on system features and client characteristics.

Although individuals with IDD have higher hospital readmission rates than the general population (Axmon et al. 2019; Reichard et al. 2019), the role of nursing in these readmissions has not been explored. In this study, the primary outcome of interest was hospital readmission. The purpose of the study was to examine the association between variation in nurse staffing levels across hospitals and 30‐day and 60‐day readmission rates among patients with IDD.

## 2 Methods

This cross‐sectional correlational investigation used secondary data from three 2016 datasets representing hospitals and the patients in them in four states: California, Florida, New Jersey, and Pennsylvania. The four states were part of a parent study. The American Hospital Association (AHA) Annual Survey, the RN4CAST‐US nurse survey, and state patient hospital discharge summaries were all connected by a common hospital identifier. The linkage was used to determine the association between hospital nurse staffing levels and readmissions among hospitalized patients with IDD, after adjusting for patient and hospital confounders. The unit of analysis for the predictor variable was the hospital and for the outcome variable was the patient. This study was approved by the XX Institutional Review Board.

### 2.1 Hospitals and Nurses

The 2016 American Hospital Association Annual Survey included hospital attributes such as state, teaching status, number of beds, and technological level. The RN4CAST‐US survey provided nurse responses, which were aggregated (calculating an average value) to the hospital level. Using addresses provided by the state boards of nursing, a modified Dillman technique was utilized to disseminate questionnaires by U.S. mail (Dillman et al. 2014). A 29% response rate was obtained from a 30% random sample of licensed registered nurses in Pennsylvania, Florida, New Jersey, and California. The respondents' employing hospitals represented more than 97% of the hospitals in those four states. A second, random sample of 1400 nonrespondents was used to address concerns about nonresponse bias. High incentives achieved an 87% response rate. Between the first and second samples, there was no discernible response bias. More information regarding the RN4CAST‐US survey has been published elsewhere (Lasater et al. 2019). At least one patient with IDD and five nurse respondents (see below) were required to include a hospital. The final sample included 595 acute care, nonfederal hospitals and the patients with IDD in Pennsylvania, Florida, New Jersey, and California. Federal hospitals, including VA hospitals and military hospitals, were excluded from the sample.

### 2.2 Patients With IDD

The study employed deidentified patient claims information from inpatient discharge summaries from 2016. The data were obtained from state sources, including the Pennsylvania Health Care Cost Containment Council, the Florida Agency for Health Care and Administration, the California Office of Statewide Health Planning and Development, and the New Jersey Department of Health and Senior Services (French et al. 2022). These states were chosen because over 90% of all patients in the four states and more than 27% of all patients in the USA are discharged by these hospitals, which account for more than 20% of all acute care hospitals nationwide (Sloane et al. 2018). Data from these state sources could be successfully linked to hospital‐specific nurse survey data.

The Centers for Medicare and Medicaid Services Chronic Conditions Data Warehouse Other Chronic Health, Mental Health, and Potentially Disabling Conditions (Table 1) provided guidance on the use of patient ICD‐10 diagnostic codes to identify patients with IDD in the claims data (Centers for Medicare and Medicaid Services 2013‐2024). Diagnosis‐Related Groups (DRG), ICD‐10 codes, primary and secondary diagnosis and procedure codes, admission and discharge dates, comorbidities, and demographic information such as age and gender were included in the deidentified data set. The patient population included for analysis was any hospitalized patient with IDD age 18 and older that was discharged alive. Multiple admissions for the same patient were identified and included in nested models. Those readmitted to different hospitals were not nested.

**Table 1:** The centers for medicare and medicaid services chronic conditions data warehouse other chronic health, mental health, and potentially disabling conditions ICD‐10 Codes for identifying IDD patients.

Description	ICD‐10 diagnosis codes
Intellectual disabilities and related conditions	DX E78.71, E78.72, F70, F71, F72, F73, F78, F78.A1, F78.A9, F79, P04.3, Q86.0, Q87.1, Q87.11, Q87.19, Q87.2, Q87.3, Q87.5, Q87.81, Q87.89, Q89.7, Q89.8, Q90.0, Q90.1, Q90.2, Q90.9, Q91.0, Q91.1, Q91.2, Q91.3, Q91.4, Q91.5, Q91.6, Q91.7, Q92.0, Q92.1, Q92.2, Q92.5, Q92.61, Q92.62, Q92.7, Q92.8, Q92.9, Q93.0, Q93.1, Q93.2, Q93.3, Q93.4, Q93.5, Q93.51, Q93.59, Q93.7, Q93.81, Q93.88, Q93.89, Q93.9, Q95.2, Q95.3, Q99.2
Autism spectrum disorders	DX F84.0, F84.3, F84.5, F84.8, F84.9
Other developmental delays	DX F81.9, F82, F88, F89

### 2.3 Patient Outcomes

Readmissions (30‐ and 60‐day) were identified if a patient was readmitted to a hospital within either 30 days or 60 days of discharge. We excluded patients who died during hospitalization (*n *= 9).

### 2.4 Nurse Staffing Levels

Via survey, hospital‐employed nurses reported the number of patients assigned to them on their last shift. Nurse staffing level was the number of patients each nurse reported having been responsible for during their last shift. The hospital‐level measure of nurse staffing level used for analysis was created by aggregating responses across nurses working within the same hospitals (Lasater et al. 2021). Per an earlier study (Lasater et al. 2021), a minimum of five nurses per hospital were utilized to ensure appropriate aggregate metrics. When the intraclass correlation (ICC) is higher than 0.60, aggregated nurse data are considered credible and generalizable (Estabrooks et al. 2011); the ICC for the data in the current study was 0.80. Regardless of nursing unit type or shift (day, evening, or night), the mean number of patients for all nurses was used to calculate hospital‐level nurse staffing. This staffing metric is better than those from administrative databases, which typically contain nurses who do not provide inpatient bedside nursing care. Nurse staffing levels were measured across the entire hospital because patients often receive nursing care on multiple nursing units of a hospital and because to date no studies have found that specialty‐specific staffing improves patient outcomes (Needleman et al. 2001). Prior research regarding nursing self‐report of workload has demonstrated predictive validity (Aiken et al. 2010).

### 2.5 Patient Characteristics

To account for patient differences that may impact outcomes, we included two different propensity scores to adjust for patient risk, including comorbidities, reasons for admission, age, and gender. Separate propensity score models were developed for the 30‐day and 60‐day readmission outcomes. The comorbidities included those described by Elixhauser et al. (1998). We also included DRG to account for variation across patients in the reasons for hospitalization. The risk adjustment was intended to control for these differences, which could confound our estimation of the association between staffing and readmission (Lane‐Fall and Neuman 2013).

### 2.6 Hospital Characteristics

Hospital size (in terms of number of beds), teaching status, and level of technology were among the hospital features measured. Small (100 or fewer beds), medium (101 to 250 beds), and large (more than 250 beds) were the three categories of hospital size. The three categories of teaching status were minor teaching (1:4 or less trainee‐to‐bed ratio), major teaching (more than 1:4 trainee‐to‐bed ratio), and nonteaching (no medical residents or fellows). If a hospital had the capacity for major organ transplants, open heart surgery, or both, it was classified as high technology.

### 2.7 Statistical Analysis

Sample‐size estimation formulas and estimated power were derived based on a method by Diggle that accounts for hierarchical models (Diggle 2002). A sample with 595 hospitals and a total of 28,446 patients nested within hospitals has 80% power to detect an odds ratio as small as 1.097 to be statistically significant at the 0.05 level assuming the overall 30‐day readmission rate is 17.1% and an ICC of 0.06 based on current data set from our group was computed using PASS2024, version 24.0.2 (NCSS LLC 2024). The hospital sample characteristics are described as percentages. Nurse staffing level was described as a continuous variable. The association of nurse staffing level with both 30‐day and 60‐day readmission was estimated both before and after controlling for hospital and patient characteristics. Hospital characteristics controlled for included size, teaching status, and technology. Patient characteristics included age, gender, comorbidities, and DRG, utilizing the propensity score. Logistic regression models were estimated to determine the association between nurse staffing level and readmission status, while adjusting for the correlation among repeated observations within patients nested within hospitals using the Generalized Linear Mixed Models (GLMM) using STAT Version 18 (STATA 2021).

## 3 Results

Table 2 presents descriptive statistics for 595 acute care, nonfederal hospitals from Pennsylvania, Florida, New Jersey, and California. The majority of hospitals were medium or large teaching hospitals without advanced technologies. Most hospitals were in California, followed by Florida, Pennsylvania, and New Jersey.

**Table 2:** Hospital characteristics.

	All hospitals	All hospitals nurse staffing
	No. (%)	M (SD)
Average patient‐to‐nurse ratio	—	4.74 (0.99)
Number of beds		
≤ 100	88 (14.8)	4.97 (1.47)
101–250	263 (44.2)	4.96 (1.10)
> 250	244 (41.0)	4.65 (0.79)
Teaching status		
None	267 (44.9)	4.92 (1.07)
Minor	283 (47.6)	4.79 (1.05)
Major	45 (7.6)	4.67 (1.05)
Technology status		
Low	315 (52.9)	5.05 (1.20)
High	280 (47.1)	4.84 (0.80)
State		
California	243 (40.8)	4.30 (0.93)
New Jersey	61 (10.3)	5.31 (0.90)
Pennsylvania	132 (22.2)	5.32 (1.06)
Florida	159 (26.7)	5.08 (1.06)

*Note:* (*n* = 595). Hospital characteristics outlining categories of beds, teaching status, and states reflect categories chosen in a prior paper using the same state and hospital data (Lasater and Mchugh 2016). Totals may not equal 100% due to rounding.

Abbreviations: M, mean; SD, standard deviation; *n,* number.

We included 39,558 hospital stays for 28,446 patients with IDD aged ≥ 18 in this study (Table 3). The average patient was 46 years old. Three‐fifths were male. Patient demographics were the same in the sample of 28,446 patients as in the 39,558 admissions. The top ten DRGs included psychosis (34.9%), septicemia combined categories (30.2%), seizures combined categories (14.7%), respiratory infections (5.2%), esophagitis, gastroenteritis, and miscellaneous digestive disorders (4.2%), cellulitis (3.8%), pneumonia and pleurisy (3.7%), and kidney and UTI (3.7%).

**Table 3:** Descriptive characteristics of patient sample: Mean (SD) or frequency (%).

Adult IDD patient demographics (*n* = 28,446 patients)	
Age (years), mean (SD)	46.0 (17.7)
Sex	
Female	11,289 (42.1%)
Male	15,553 (57.9%)
**Top 10 DRGs,** ***n*** **(%)** (*n* = 39,558 admissions)	
885 Psychoses	5555 (34.6%)
871 Septicemia without mechanical ventilation (MV) > 96 h with MMC	3904 (24.3%)
101 Seizures without MMC	1655 (10.3%**)**
872 Septicemia or severe sepsis without mechanical ventilation (MV)	941 (5.9%)
177 Respiratory infections and inflammations with MCC/CC	836 (5.2%)
100 Seizures with MMC	706 (4.4%)
392 Esophagitis, gastroenteritis and miscellaneous digestive disorders without MCC	669 (4.2%)
603 Cellulitis without MMC	612 (3.8%)
194 Simple Pneumonia and pleurisy with CC	600 (3.7%)
690 Kidney and UTI without MMC	566 (3.5%)
**Outcomes** (*n* = 39,558 admissions)	
30‐Day Readmission	6775 (17.1%)
60‐Day Readmission	9386 (23.7%)

*Note:* Totals may not equal 100% due to rounding

Abbreviations: CC, complication or comorbidity; MCC, major complication or comorbidity; *n*, number; SD, standard deviation.

The readmission rate was 17.1% (30‐day) and 23.7% (60‐day). The average nurse staffing level across hospitals was 4.7 patients per nurse (SD of 0.99). Nurse staffing levels were best (i.e., fewer patients per nurse) in large hospitals compared to small and medium‐sized hospitals. Staffing was also better at major teaching hospitals compared to minor and nonteaching hospitals. Nurses in high technology hospitals had more patients per nurse than low technology. California had the best nurse staffing levels, followed by Florida, New Jersey, and Pennsylvania. This difference was one patient per nurse comparing California to Pennsylvania.

Every patient added to the nurse's workload was associated with 7% higher odds on 30‐day readmission in both the unadjusted and adjusted regression models (Table 4). Every patient added to the nurse's workload was associated with 8% higher odds on 60‐day readmission in the unadjusted model, and in the adjusted model, every patient added to the nurse's workload was associated with 9% higher odds on 60‐day readmission (Table 5).

**Table 4:** Associations of nurse staffing levels with 30‐day readmissions.

Patient outcomes	OR	95% CI		*p*
		*LL*	*UL*	
Model 1: unadjusted 30‐day readmissions				
Nurse staffing level	1.07	1.02	1.11	0.002
Model 2: adjusted 30‐day readmissions				
Nurse staffing level	1.07	1.11	1.93	0.001
Bed size				
< 100	Ref.	Ref.	Ref.	Ref.
101−250	0.94	0.782	1.13	0.527
> 250	1.03	0.850	1.25	0.749
Teaching status				
None	Ref.	Ref.	Ref.	Ref.
Minor	1.03	0.946	1.13	0.467
Major	1.56	1.01	1.32	0.034
Technology status				
Low	Ref.	Ref.	Ref.	Ref.
High	0.91	0.824	1.00	0.058
Risk propensity score	2.39	1.63	3.51	< 0.001

*Note:* Hospital characteristics include bed size, teaching status, technology status, and state. Propensity score risk adjusts for patient characteristics, including comorbidities, reason for admission, age, and gender.

Abbreviations: CI, confidence interval; LL, lower limit; OR, odds ratio; UL, upper limit.

Unadjusted and adjusted generalized linear mixed models.

**Table 5:** Associations of nurse staffing levels with 60‐day readmissions.

Patient outcomes	OR	95% CI		*p*
		*LL*	*UL*	
Model 1: unadjusted 60‐day readmissions				
Nurse staffing level	1.08	1.04	1.12	< 0.001
Model 2: adjusted 60‐day readmissions				
Nurse staffing level	1.09	1.04	1.13	< 0.001
Bed size				
Bed size < 100	Ref.	Ref.	Ref.	Ref.
Bed size 101−250	0.93	0.783	1.11	0.435
Bed size > 250	1.05	0.872	1.26	0.613
Teaching status				
Teaching status none	Ref.	Ref.	Ref.	Ref.
Teaching status minor	1.04	0.960	1.14	0.312
Teaching status major	1.11	0.977	1.27	0.109
Technology status				
Technology status low	Ref.	Ref.	Ref.	Ref.
Technology status high	0.90	0.821	0.991	0.033
Risk propensity score	133.0	85.8	206.1	< 0.001

*Note:* Hospital characteristics include bed size, teaching status, technology status, and state. Propensity score risk adjusts for patient characteristics, including comorbidities, reason for admission, age, and gender.

Abbreviations: CI, confidence interval; LL, lower limit; OR, odds ratio; UL, upper limit.

Unadjusted and adjusted generalized linear mixed models.

## 4 Discussion

In this study of 39,558 hospital stays for 28,446 adults with IDD across 595 hospitals in four large U.S. states, we aimed to test associations between nurse staffing levels and 30‐ and 60‐day readmissions. Consistent with literature in other populations, we found that better nurse staffing levels were associated with lower odds for readmission. This study reinforces that ensuring adequate nurse staffing levels for inpatients with IDD—a population requiring some of the most intensive nursing care—is associated with lower odds of both 30‐ and 60‐day readmission (7% and 9%, respectively in the adjusted models).

To our knowledge, this is the first study to evaluate the association between nurse staffing levels and hospital readmission rates in adults with IDD, and, therefore, comparisons in the literature are not available. Some literature reveals disproportionate effects of nurse staffing levels on vulnerable populations who, like those with IDD, may benefit from greater intensity of nursing care. Examples of these populations include those with Alzheimer's disease and related dementia, substance use disorder, or mental illness (French et al. 2022; Kumar et al. 2022; Kutney‐Lee and Aiken 2008; White et al. 2018). Despite the challenges posed in the care of patients with IDD, nurses aim to meet the complex care needs of their patients (Appelgren et al. 2018), and appropriate staffing helps to enable that care.

Two studies examined nurse staffing and readmission as we did. The average nurse staffing level in our study was 4.74 patients per nurse (SD: 0.99), with a 60‐day readmission rate of 23.7% for patients with IDD. Each additional patient‐per‐nurse was associated with 9% higher 60‐day readmission odds. These figures align closely with findings from Lasater et al. (2021), who reported a medical‐surgical nurse staffing ratio of 6.3 (SD: 1.0) and a 60‐day readmission rate of 23% in a national sample of patients hospitalized with sepsis. In the Lasater study, each additional patient‐per‐nurse equated to 7% higher odds of 60‐day readmission, a result quite like ours. This similarity is notable, as sepsis patients, like many patients with IDD, often present with complex care needs that require more nursing support. Our results also mirror those of French et al. (2022), who found that among surgical patients with opioid use disorder, an additional patient per nurse was associated with 9% lower odds of 30‐day readmission. In this population, the readmission rate was 19% and the nurse staffing level was 4.3 (SD: 0.9).

The small but meaningful drop in readmissions (7%–9%) is clinically meaningful to patients with IDD and to nurses who care for them based on practical importance, while it suggests substantial cost savings and reduced penalties for hospitals. This figure also reflects improvements in discharge planning, patient outcomes, and more effective follow‐up care, indicating that hospital interventions may be working as intended. Fewer readmissions free up hospital beds, improve capacity management and allow resources to be redirected toward new patients rather than readmitted ones. Lower readmission rates often correlate with higher patient satisfaction since patients prefer to avoid repeat hospitalizations. Many healthcare insurers aim to reduce readmissions as an indicator of value‐based care, and improvements in this metric may signal progress in aligning these policies. Even a modest decrease in readmissions can have meaningful implications, particularly for patients with IDD, whose baseline readmission rates are substantially higher than patients without IDD. Nurses serve as a surveillance safety net for critically ill patients and offer around‐the‐clock bedside care. To ensure that nurses can efficiently and reliably manage patient care needs, coordinate care, anticipate clinical deterioration, avoid injury, and educate patients and families, it is critical to have a sufficient number of nurses with a manageable workload. There is substantial evidence spanning decades worldwide, suggesting patients treated in hospitals with lower nurse staffing levels (i.e., more patients per nurse) experience worse outcomes (i.e., death, adverse events, and readmissions) than patients treated in hospitals with higher nurse staffing levels. In 2018, the International Council of Nurses issued a safe staffing position statement in response, urging governments and nursing organizations to implement evidence‐based nurse staffing practices. Despite this, minimum nurse staffing standards are rarely put into practice. Only California and Oregon in the United States and the state of Victoria in Australia, Wales, and Ireland abroad have implemented similar legislation (McHugh et al. 2020). Although there are no universally accepted recommended staffing levels, two U.S. states have legislated 5 patients per nurse on medical/surgical units (California Legislative Information 1999). Our findings are consistent with this value, although our sample included nurses from various hospital departments rather than exclusively medical‐surgical units.

Proper nursing care can facilitate a reduction in readmissions (Aiken et al. 2010; Lasater and Mchugh 2016). In 2018, the average hospital 30‐day readmission rate among U.S. adults averaged 14% (Weiss and Jiang 2018), while our study of patients with IDD had a rate of 17.7% for 30‐day readmissions, which is 27% higher by comparison. The higher readmission rate suggests that the healthcare system may not be adequately equipped to address the unique needs of patients with IDD, potentially reflecting issues in the structure and processes of care delivery for this population. Patients with IDD have specific health vulnerabilities and a higher prevalence of comorbidities, such as heart disease, diabetes, and mental health conditions like psychosis that can impact their outcomes (Reppermund et al. 2020). Although psychosis was accounted for using a propensity score in our study, the 34% principal diagnosis rate for psychosis illustrates the burden of mental health comorbidities (Balogh et al. 2018).

These results support our chosen framework (QHOM), which emphasizes the dynamic relationships between system characteristics, client factors, interventions, and outcomes. The QHOM recognizes that client characteristics interact with interventions and system factors, which influence outcomes. The QHOM posits that interventions do not directly produce outcomes but are mediated by both system and client characteristics. Specifically, the study demonstrates that a systems intervention like nurse staffing may reduce readmission rates for a subgroup of patients with IDD. Our findings show the model's relevance, highlighting the modest yet statistically significant association of nurse staffing with odds of readmissions for those with IDD. Focusing on improving nurse staffing is a systems‐based solution that is expected to improve outcomes for all patients (Lasater and Mchugh 2016) but especially for patients with IDD.

Hospital and nursing administrators should therefore prioritize the complex and often higher care needs of patients with IDD when assigning nurse staffing levels. This managerial ecommendation acknowledges that patients with IDD may require more time, specialized attention, and resources compared to typical patients, ensuring better care outcomes and patient safety. According to a systematic review, nurses who provided nursing care for hospitalized patients with IDD reported that they were undertrained, needed more time to finish nursing care, struggled with erratic patient behavior, had trouble communicating with family caregivers, and experienced difficulty with interdisciplinary communication (Appelgren et al. 2018). Providing the nurse staffing levels needed to meet these demands may also offset nurses' moral distress, emotional burnout, job dissatisfaction, safety and quality concerns as well as costly turnover (McHugh et al. 2020).

Some scholars have proposed that expanded training and education could help nurses provide better care for people with IDD and reduce poor outcomes (Appelgren et al. 2018; Redley et al. 2019). The training, however, is unlikely to prepare nurses for patients with both IDD and mental health comorbidities (i.e., psychosis) which were prominent in our sample. In evaluating the UK's Equality Act of 2010, which pertains to patients with IDD, only half of medical practitioners reported making “reasonable adjustments,” leading the authors to call for improved medical education (Redley et al. 2019). While these interventions may be beneficial in the right context and with sufficient resources, they fail to address the system‐level hindrances that prevent nurses from providing patient‐centered care in the first place (Brooks Carthon et al. 2021). This study highlights the potential of system interventions, such as improving the nurse staffing ratio, as an approach to reducing readmissions and enhancing care for patients with IDD.

Our findings also have economic and quality‐of‐care implications. The CMS penalty structures, like the Hospital Readmissions Reduction Program, can have both positive and negative economic and quality of care effects on hospitals (Psotka et al. 2020). For example, hospitals with high readmission rates face payment reductions from CMS, which affects their revenue. The prospect of penalties can encourage hospitals to design programs and strategies aimed at reducing readmissions, which can lead to long‐term cost savings. Moreover, private insurers may use this program as a model for reducing readmissions (Ayabakan et al. 2021). This financial pressure, however, can also lead to unintended consequences. Hospitals may focus on reducing readmissions for patients who are easier to manage, potentially exacerbating disparities in care for more vulnerable populations (Hawn 2015).

The use of 2016 data in this study raises the question about the generalizability and contemporary relevance of our findings. Since that time, there may have been significant changes in hospital policies, nurse staffing patterns, or care delivery models that could influence readmission rates. To our knowledge of the field, however, hospital policies and care delivery models for this population have not changed in the ensuing years. Notably, the COVID‐19 pandemic placed unprecedented strain on nursing resources. The American Association of Critical Care Nurses reports that nurse staffing was “poor” after the pandemic (Blake 2022). Nurse staffing levels continue to face challenges and have prompted the development of new and creative nurse staffing arrangements (Weberg 2023). Additionally, post‐COVID changes in community care may contribute to longer hospital stays and difficulties with patient placement. Also, some adults with IDD may now be boarded in emergency or outpatient settings rather than being admitted for fear of losing their residential placements. The link we observed between nurse staffing and readmission may be even more pronounced today and warrants further investigation in the current context.

### 4.1 Limitations

The cross‐sectional design limits our ability to make causal inferences about the association between nurse staffing and readmissions. Another limitation is that claims data likely do not identify all patients with IDD, particularly if functional impairments are minimal. Thus, our results may not generalize to the population with IDD that presents with limited impairment. Although individual nurses cannot be linked to individual patients, our focus is on organizational‐level constructs, comparing organizational constructs across the hospital sample (Dierkes et al. 2021). Nursing is studied as a hospital‐level resource. Furthermore, patients often receive care from numerous nurses throughout different hospital units over the course of their hospital stay. Other common predictors of readmission for people with IDD including communication needs, behavioral support needs, and residential environments, were not accounted for given lack of data. The combination of mental health comorbidities and IDD results in more complex health needs, often leads to poor outcomes, including more frequent readmissions but has not been studied in relation to nurse staffing (Balogh et al. 2018). The exclusion of federal hospitals, such as VA hospitals and military hospitals, which may include individuals with IDD, from the sample limits the generalizability of our findings to these settings. While this study used 2016 data, we do not expect that the use of newer data would change the findings, which reflect associations between staffing levels and readmissions for those with IDD. In the future, we do anticipate replicating the study when the unique combination of nurse workforce and corresponding patient IDD data becomes available.

## 5 Conclusions

Over 20 years ago, the Institute of Medicine urged the advancement of system‐level improvements to patient care (Institute of Medicine and Committee on Quality of Health Care in America 2000). Since then, various patient populations have been shown to have improved outcomes when cared for in hospitals with better nurse staffing levels (Brooks Carthon et al. 2021; Lasater and Mchugh 2016). This study shows that those with IDD appear to benefit from increased nurse staffing levels as demonstrated through significantly reduced 30‐ and 60‐day readmissions. The consequences of readmission for patients with IDD, their families, and the healthcare system warrant system‐level attention to nurse staffing levels, as identified herein.

## Author Contributions

**Lynne Moronski:** conceptualization, data curation, formal analysis, project administration, methodology, validation, visualization, writing – original draft preparation, reviewing and editing, **Eileen Lake:** conceptualization, formal analysis, supervision, methodology, validation, visualization, writing – original draft preparation, reviewing and editing, **Matthew McHugh:** conceptualization, formal analysis, supervision, methodology, validation, visualization, writing – original draft preparation, reviewing and editing.

## Conflicts of Interest

The authors declare no conflicts of interest.

## Data Availability

The data that support the findings of this study are available on request from the corresponding author. The data are not publicly available due to privacy or ethical restrictions.

## References

[nur70013-bib-0001] Aiken, L. H. 2002. “Hospital Nurse Staffing and Patient Mortality, Nurse Burnout, and Job Dissatisfaction.” Journal of the American Medical Association 288, no. 16: 1987–1993. 10.1001/jama.288.16.1987. 12387650

[nur70013-bib-0002] Aiken, L. H. , D. M. Sloane , J. P. Cimiotti , et al. 2010. “Implications of the California Nurse Staffing Mandate for Other States.” Health Services Research 45, no. 4: 904–921. 10.1111/j.1475-6773.2010.01114.x. 20403061 PMC2908200

[nur70013-bib-0003] Ailey, S. H. , T. Johnson , L. Fogg , and T. R. Friese . 2014. “Hospitalizations of Adults With Intellectual Disability in Academic Medical Centers.” Intellectual and Developmental Disabilities 52, no. 3: 187–192. 10.1352/1934-9556-52.3.187. 24937744

[nur70013-bib-0004] American Association on Intellectual and Developmental Disabilities . 2025. Defining Criteria for Intellectual Disability. https://www.aaidd.org/intellectual-disability/definition.

[nur70013-bib-0005] Appelgren, M. , C. Bahtsevani , K. Persson , and G. Borglin . 2018. “Nurses' Experiences of Caring for Patients With Intellectual Developmental Disorders: A Systematic Review Using a Meta‐Ethnographic Approach.” BMC Nursing 17: 51. 10.1186/s12912-018-0316-9. 30524202 PMC6276187

[nur70013-bib-0006] Axmon, A. , M. Björkman , and G. Ahlström . 2019. “Hospital Readmissions Among Older People With Intellectual Disability in Comparison With the General Population.” Journal of Intellectual Disability Research 63, no. 6: 593–602. 10.1111/jir.12601. 30734976 PMC6850197

[nur70013-bib-0007] Ayabakan, S. , I. Bardhan , and Z. Zheng . 2021. “Triple Aim and the Hospital Readmission Reduction Program.” Health Services Research and Managerial Epidemiology 8: 2333392821993704. 10.1177/2333392821993704. 33644257 PMC7894595

[nur70013-bib-0008] Balogh, R. , E. Lin , K. Dobranowski , A. Selick , A. S. Wilton , and Y. Lunsky . 2018. “All‐Cause, 30‐Day Readmissions Among Persons With Intellectual and Developmental Disabilities and Mental Illness.” Psychiatric Services 69, no. 3: 353–357. 10.1176/appi.ps.201600534. 29137556

[nur70013-bib-0009] Blake, N. 2022. “Appropriate Staffing After the Pandemic: A New Challenge for Healthy Work Environments.” AACN Advanced Critical Care 33, no. 3: 280–282. 10.4037/aacnacc2022746. 36067256

[nur70013-bib-0010] Brooks Carthon, J. M. , L. Hatfield , H. Brom , et al. 2021. “System‐Level Improvements in Work Environments Lead to Lower Nurse Burnout and Higher Patient Satisfaction.” Journal of Nursing Care Quality 36, no. 1: 7–13. 10.1097/NCQ.0000000000000475. 32102025 PMC7483185

[nur70013-bib-0011] California Legislative Information . 1999. Assembly Bill 394 Analytics. http://www.leginfo.ca.gov/pub/99-00/bill/asm/ab_0351-0400/ab_394_cfa_19990708_164003_sen_comm.html.

[nur70013-bib-0012] Centers for Disease Control and Prevention . 2024. Developmental Disability Basics. https://www.cdc.gov/child-development/about/developmental-disability-basics.html.

[nur70013-bib-0013] Centers for Medicare and Medicaid Services . 2013‐2024. Chronic Conditions Data Warehouse Other Chronic Health, Mental Health, and Potentially Disabling Conditions. https://www2.ccwdata.org/web/guest/condition-categories-other.

[nur70013-bib-0014] Dierkes, A. M. , L. H. Aiken , D. M. Sloane , and M. D. McHugh . 2021. “Association of Hospital Nursing and Postsurgical Sepsis.” PLoS One 16, no. 10: e0258787. 10.1371/journal.pone.0258787. 34662355 PMC8523045

[nur70013-bib-0015] Diggle, P. 2002. Analysis of Longitudinal Data. Oxford university press.

[nur70013-bib-0016] Dillman, D. A. , J. D. Smyth , and L. M. Christian . 2014. Internet, Phone, Mail, and Mixed‐mode Surveys: The Tailored Design Method (4th ed. Wiley.

[nur70013-bib-0017] Elixhauser, A. , C. Steiner , D. R. Harris , and R. M. Coffey . 1998. “Comorbidity Measures for Use With Administrative Data.” Medical Care 36, no. 1: 8–27. 10.1097/00005650-199801000-00004. 9431328

[nur70013-bib-0018] Ervin, D. A. , and J. Merrick . 2014. “Intellectual and Developmental Disability: Healthcare Financing.” Frontiers in Public Health 2: 160. 10.3389/fpubh.2014.00160. 25309896 PMC4173221

[nur70013-bib-0019] Escudé, C. 2022. “Advancing Health Equity For People With Intellectual and Developmental Disabilities.” Health Affairs Forefront 10: 1377. 10.1377/forefront.20221019.4166.

[nur70013-bib-0020] Estabrooks, C. A. , W. K. Midodzi , G. G. Cummings , K. L. Ricker , and P. Giovannetti . 2011. “The Impact of Hospital Nursing Characteristics on 30‐day Mortality.” JONA: The Journal of Nursing Administration 41, no. 7/8: S58–S68. 10.1097/00006199-200503000-00002. 21799356

[nur70013-bib-0021] French, R. , M. D. McHugh , L. H. Aiken , P. Compton , S. H. Meghani , and J. M. Brooks Carthon . 2022. “Nursing Resources Linked to Postsurgical Outcomes for Patients With Opioid Use Disorder: An Observational Study.” Annals of Surgery Open 3, no. 3: e185. 10.1097/AS9.0000000000000185. 36199489 PMC9508985

[nur70013-bib-0022] Haegdorens, F. , P. Van Bogaert , K. De Meester , and K. G. Monsieurs . 2019. “The Impact of Nurse Staffing Levels and Nurse's Education on Patient Mortality in Medical and Surgical Wards: An Observational Multicentre Study.” BMC Health Services Research 19, no. 1: 864. 10.1186/s12913-019-4688-7. 31752859 PMC6868706

[nur70013-bib-0024] Hawn, M. T. 2015. “Unintended Consequences of the Hospital Readmission Reduction Program.” Annals of Surgery 261, no. 6: 1032–1033. 10.1097/SLA.0000000000001150. 25647062

[nur70013-bib-0062] Heslop, P. , S. Turner , S. Read , J. Tucker , S. Seaton , and B. Evans . 2019. “Implementing Reasonable Adjustments for Disabled People in Healthcare Services.” Nursing Standard 34, no. 8: 29–34. 10.7748/ns.2019.e11172 31468776

[nur70013-bib-0025] Hosking, F. J. , I. M. Carey , S. DeWilde , T. Harris , C. Beighton , and D. G. Cook . 2017. “Preventable Emergency Hospital Admissions Among Adults With Intellectual Disability in England.” Annals of Family Medicine 15, no. 5: 462–470. 10.1370/afm.2104. 28893817 PMC5593730

[nur70013-bib-0026] Institute of Medicine and Committee on Quality of Health Health Care in America . 2000. To Err Is Human: Building a Safer Health System, edited by L. T. Kohn , J. M. Corrigan , and M. S. Donaldson . US: National Academies Press. 10.17226/9728. 25077248

[nur70013-bib-0027] Integrity Inc . 2019. What Is the Difference Between Developmental and Intellectual Disabilities? https://www.integrityinc.org/what-is-the-difference-between-developmental-and-intellectual-disabilities/.

[nur70013-bib-0029] Kumar, A. , D. Sloane , L. Aiken , and M. McHugh . 2022. “Hospital Nursing Factors Associated With Decreased Odds of Mortality in Older Adult Medicare Surgical Patients With Depression.” BMC Geriatrics 22, no. 1: 665. 10.1186/s12877-022-03348-1. 35963991 PMC9375432

[nur70013-bib-0030] Kutney‐Lee, A. , and L. H. Aiken . 2008. “Effect of Nurse Staffing and Education on the Outcomes of Surgical Patients With Comorbid Serious Mental Illness.” Psychiatric Services 59, no. 12: 1466–1469. 10.1176/ps.2008.59.12.1466. 19033176 PMC2596648

[nur70013-bib-0031] Lane‐Fall, M. B. , and M. D. Neuman . 2013. “Outcomes Measures and Risk Adjustment.” International Anesthesiology Clinics 51, no. 4: 10–21. 10.1097/AIA.0b013e3182a70a52. 24088885 PMC3816010

[nur70013-bib-0032] Lasater, K. B. , O. F. Jarrín , L. H. Aiken , M. D. McHugh , D. M. Sloane , and H. L. Smith . 2019. “A Methodology for Studying Organizational Performance: A Multistate Survey of Front‐Line Providers.” Medical Care 57, no. 9: 742–749. 10.1097/MLR.0000000000001167. 31274782 PMC6690788

[nur70013-bib-0033] Lasater, K. B. , and M. D. Mchugh . 2016. “Nurse Staffing and the Work Environment Linked to Readmissions Among Older Adults Following Elective Total Hip and Knee Replacement.” International Journal for Quality in Health Care 28, no. 2: 253–258. https://www.ncbi.nlm.nih.gov/pmc/articles/PMC4833205/pdf/mzw007.pdf. 26843548 10.1093/intqhc/mzw007 PMC4833205

[nur70013-bib-0034] Lasater, K. B. , D. M. Sloane , M. D. McHugh , et al. 2021. “Evaluation of Hospital Nurse‐to‐Patient Staffing Ratios and Sepsis Bundles on Patient Outcomes.” American Journal of Infection Control 49, no. 7: 868–873. 10.1016/j.ajic.2020.12.002. 33309843 PMC8190185

[nur70013-bib-0036] Lindgren, S. , E. Lauer , E. Momany , et al. 2021. “Disability, Hospital Care, and Cost: Utilization of Emergency and Inpatient Care by a Cohort of Children With Intellectual and Developmental Disabilities.” Journal of Pediatrics 229: 259–266. 10.1016/j.jpeds.2020.08.084. 32890584 PMC7885996

[nur70013-bib-0037] Ma, C. , J. Shang , and P. Stone . 2014. “Can Nurse Work Environment Influence Readmission Risk? A Systematic Review.” Nursing: Research and Reviews 4: 91–101. 10.2147/NRR.S46156.

[nur70013-bib-0038] McHugh, M. D. , L. H. Aiken , D. M. Sloane , C. Windsor , C. Douglas , and P. Yates . 2021. “Effects of Nurse‐to‐Patient Ratio Legislation on Nurse Staffing and Patient Mortality, Readmissions, and Length of Stay: A Prospective Study in a Panel of Hospitals.” Lancet 397, no. 10288: 1905–1913. 10.1016/S0140-6736(21)00768-6. 33989553 PMC8408834

[nur70013-bib-0039] McHugh, M. D. , L. H. Aiken , C. Windsor , C. Douglas , and P. Yates . 2020. “Case for Hospital Nurse‐to‐Patient Ratio Legislation in Queensland, Australia, Hospitals: An Observational Study.” BMJ Open 10, no. 9: e036264. https://bmjopen.bmj.com/content/bmjopen/10/9/e036264.full.pdf. 10.1136/bmjopen-2019-036264 PMC7476482 32895270

[nur70013-bib-0040] Mitchell, P. H. , S. Ferketich , and B. M. Jennings , & American Academy of Nursing Expert Panel on Quality Health Care. 1998. “Quality Health Outcomes Model.” Image‐‐the Journal of Nursing Scholarship 30, no. 1: 43–46. 9549940 10.1111/j.1547-5069.1998.tb01234.x

[nur70013-bib-0061] Moloney, M. , T. Hennessy , and O. Doody . 2021. “Reasonable Adjustments for People With Intellectual Disability in Acute Care: A Scoping Review of the Evidence.” BMJ Open 11, no. 2: e039647. 10.1136/bmjopen-2020-039647 PMC7903074 33619184

[nur70013-bib-0042] NCSS LLC . 2024. *PASS 2024 Power Analysis and Sample Size Software* In. ncss.com/software/pass.

[nur70013-bib-0043] Needleman, J. , P. Buerhaus , S. Mattke , M. Stewart , and K. Zelevinsky . 2001. “Nurse Staffing and Patient Outcomes in Hospitals.” In Harvard School of Public Health Boston.

[nur70013-bib-0045] Perez‐Stable, E. , and R. Valdez . 2023. Announcement of Decision to Designate People With Disabilities as a Population With Health Disparities. https://nimhd.nih.gov/about/directors-corner/messages/health-disparities-population-designation.html.

[nur70013-bib-0046] Psotka, M. A. , G. C. Fonarow , L. A. Allen , et al. 2020. “The Hospital Readmissions Reduction Program.” JACC: Heart Failure 8, no. 1: 1–11. 10.1016/j.jchf.2019.07.012. 31606360

[nur70013-bib-0060] Redley, M. , I. Lancaster , A. Pitt , et al. 2019. “‘Reasonable Adjustments’ Under the UK's Equality Act 2010: An Enquiry Into the Care and Treatment to Patients With Intellectual Disabilities in Acute Hospital Settings.” Journal of Applied Research in Intellectual Disabilities 32, no. 6: 1412–1420. 10.1111/jar.12623. 31218787 PMC8045550

[nur70013-bib-0047] Reichard, A. , E. Haile , and A. Morris . 2019. “Characteristics of Medicare Beneficiaries With Intellectual or Developmental Disabilities.” Intellectual and Developmental Disabilities 57, no. 5: 405–420. 10.1352/1934-9556-57.5.405. 31568735

[nur70013-bib-0049] Reppermund, S. , P. Srasuebkul , K. Dean , and J. N. Trollor . 2020. “Factors Associated With Death in People With Intellectual Disability.” Journal of Applied Research in Intellectual Disabilities 33, no. 3: 420–429. 10.1111/jar.12684. 31786826

[nur70013-bib-0050] Sloane, D. M. , H. L. Smith , M. D. McHugh , and L. H. Aiken . 2018. “Effect of Changes in Hospital Nursing Resources on Improvements in Patient Safety and Quality of Care: A Panel Study.” Medical Care 56, no. 12: 1001–1008. 10.1097/mlr.0000000000001002. 30363019 PMC6231998

[nur70013-bib-0051] STATA . 2021. “Stata Statistical Software Release.” In *(Version 18.0)* . StataCorp LLC.

[nur70013-bib-0053] University of Minnesota . 2024. *How Many People Have IDD?* https://risp.umn.edu/products/key-questions/how-many-people-have-idd.

[nur70013-bib-0059] Webber, R. , B. Bowers , and C. Bigby . 2010. “Hospital Experiences of Older People With Intellectual Disability: Responses of Group Home Staff and Family Members.” Journal of Intellectual and Developmental Disability 35, no. 3: 155–164. 10.3109/13668250.2010.491071. 20695830

[nur70013-bib-0054] Weberg, PhD, MHI, RN, FAAN, D. 2023. “Fractures in the Faultline: Addressable Issues in Nursing.” *Nursing Administration Quarterly* 47, no. 3: p E21–E26. 10.1097/NAQ.0000000000000581. 37261418

[nur70013-bib-0055] Weiss, A. , and H. Jiang . 2018. Overview of Clinical Conditions With Frequent and Costly Hospital Readmissions by Payer, 2018. *Healthcare Cost and Utilization Project (HCUP) Statistical Brief* . Agency for Healthcare Research and Quality. 34460186

[nur70013-bib-0056] White, E. M. , J. G. Smith , R. L. Trotta , and M. D. McHugh . 2018. “Lower Postsurgical Mortality for Individuals With Dementia With Better‐Educated Hospital Workforce.” Journal of the American Geriatrics Society 66, no. 6: 1137–1143. 10.1111/jgs.15355. 29558568 PMC6105464

[nur70013-bib-0058] Wirtz, J. , S. H. Ailey , S. Hohmann , and T. Johnson . 2020. “Patient Outcomes Associated With Tailored Hospital Programs for Intellectual Disabilities.” American Journal of Managed Care 26, no. 3: e84–e90. 10.37765/ajmc.2020.42640. 32181620

[nur70013-bib-0057] World Bank . 2023. Disability: Key Facts. https://www.who.int/news-room/fact-sheets/detail/disability-and-health#:~:text=Key%20facts,1%20in%206%20of%20us.

